# Disgraceful Advertisements

**Published:** 1844-06

**Authors:** 


*Disgraceful Advertisements.*—No better evidence of the corruption of the times and the boldness of those who live by imposition, and even crime, could be adduced, than many of the advertisements in this and all the great Atlantic cities. The advertisers seem utterly shameless, besides being thoroughly unprincipled. These shocking specimens of ingenious corruption, under the guise of Samaritan efforts for suffering humanity, are a disgrace to the newspapers, the medium through which the concentrated vileness of a mighty host of depredators on health obtains an introduction to the ignorant community.

Look at the advertiser's description of the *Portuguese Female Pills!* "The combination of ingredients of which these pills are composed, have made them the wonder and admiration of the world." "They must not be used during pregnancy, for though always mild, safe and healthy, they are certain to produce miscarriage, if used during that period." This false caution creates a positive demand for them for the most wicked purposes. And so of a multitude of others which we have neither time nor patience further to allude to. These advertisements are admitted into papers which circulate in families and lie on parlor tables, and the mischief which is done in various ways through their means is incalculable.—*Ibid.*

